# A SEIPS-Based Analysis to Understand Safety Culture During Postpartum Hemorrhage

**DOI:** 10.3390/healthcare13050499

**Published:** 2025-02-26

**Authors:** Kaitlyn L. Hale-Lopez, Madelyn M. Saenz, Neelam Verma, Shruti Chakravarthy, Rebecca Ebert-Allen, William F. Bond, Abigail R. Wooldridge

**Affiliations:** 1 Department of Industrial and Enterprise Systems Engineering, Grainger College of Engineering, University of Illinois Urbana-Champaign, Urbana, IL 61801, USA; 2 Department of Obstetrics and Gynecology, University of Illinois College of Medicine Peoria, Peoria, IL 61805, USA; 3 Jump Simulation, a Collaboration of OSF Healthcare and the University of Illinois College of Medicine, Peoria, IL 61805, USA

**Keywords:** postpartum hemorrhage, Systems Engineering Initiative for Patient Safety, safety culture, sociotechnical systems

## Abstract

**Background/Objectives:** Maternal mortality occurs at alarming rates in the United States. In 2018, there were 17 maternal deaths for every 100,000 births—double that of other high-income countries, including France and Canada. Postpartum hemorrhage (i.e., excessive blood loss during delivery or within the 24 h following) is a leading cause of maternal mortality and is a treatable condition if identified and managed in a timely manner. One aspect of work that impacts patient care during postpartum hemorrhage is the safety culture. The safety culture is the beliefs, values, and norms shared by members of the organization that influence their actions and behaviors. In this study, we use the Systems Engineering Initiative for Patient Safety (SEIPS) model to understand and describe how the sociotechnical system shapes safety culture during postpartum hemorrhage. **Methods:** We conducted interviews and focus groups with 29 clinicians to describe the work system and the barriers and facilitators during postpartum hemorrhage. Then, we inductively categorized the barriers and facilitators into emergent properties of sociotechnical systems related to safety culture. **Results:** We identified 45 barriers and 158 facilitators into five emergent properties related to the safety culture (i.e., staffing, communication, organizational management and leadership, organizational processes, and teamwork). The participants identified more positive aspects than negative, suggesting that the safety culture positively influences their actions and behaviors. **Conclusions:** Our results indicate that safety culture could be improved by redesigning the work system to mitigate barriers related to staffing, communication, organizational management, and teamwork that hinder the safety culture.

## 1. Introduction

Maternal mortality occurs at an alarming rate in the United States. In 2018, there were 17 maternal deaths for every 100,000 births—this ratio is double other high-income countries, including France and Canada [1,2]. A leading cause of maternal mortality is postpartum hemorrhage. Postpartum hemorrhage is when a patient has excessive blood loss; if within 24 h of delivery, it is considered primary postpartum hemorrhage, and if up to 12 weeks following delivery, it is considered secondary postpartum hemorrhage [3,4,5]. Postpartum hemorrhage can sometimes be prevented and, if not, can be better mitigated in treated if excessive blood loss is identified and interventions are initiated promptly [6,7]. While variation exists in international guidelines on the management of PPH [8], care bundles that incorporate three or more evidence-based best practices can improve multiple postpartum hemorrhage outcomes [9], in particular the California Maternal Quality Care Collaborative (CMQCC) [10] and E-MOTIVE bundles [11]. Evidence-based best practices to intervene in postpartum hemorrhage include early prediction or warning systems (i.e., alerts), pharmacological treatments including uterotonics (e.g., oxytocin, prostaglandins) and coagulants (e.g., tranexamic acid, fibrinogen), intravenous crystalloids, uterine massage, intrauterine devices like the Bakri balloon and the Jada device, and surgical management (e.g., laparotomy, artery ligation, hysterectomy) [9,12,13,14,15]. Identifying that a patient is at increased risk for bleeding or is already having early bleeding allows clinicians to perform interventions to prepare for and stabilize the patient prior to a severe or catastrophic postpartum hemorrhage occurring. One aspect of work that impacts patient care during postpartum hemorrhage is the safety culture.

Before defining safety culture, and how it impacts postpartum hemorrhage care, let us first examine culture as a concept. Culture is composed of observable behaviors, artifacts, articulated values, and assumptions of groups of humans [16]—in short, cultures do not exist without humans, and some have argued that humans cannot exist without culture [17]. Discourse relating culture to work spans many levels; for example, how global and national culture influences work, cultures of specific professional groups [18], as well as cultures at the organizational level [19]. Organizational culture is the shared assumptions, values, and beliefs within an organization; newcomers to the organization are socialized through enculturation and taught that these are the proper ways to think, feel, and behave [20]. Some have argued that safety culture is a specific kind or facet of organizational culture [21]. While variation in definitions exist, there is consensus that safety culture consists of the beliefs, values, and norms shared by members of the organization that influence their actions and behaviors related to and/or in pursuit of safety [22,23,24]. Safety culture has been studied across industries [21]. In health care, safety culture, or patient safety culture, is widely acknowledged as crucial to improving safety for both patients and health care workers [25,26]—importantly, improving safety culture, and the safety of patients and workers, in health care requires a systems’ perspective, rather than focusing on individuals in a piecemeal fashion [27,28].

In this paper, we present the argument that safety culture specifically, and any aspect of organizational culture in general, is part of the system in which workers perform their work. Our argument is rooted in human factors/ergonomics, which is, according to the International Ergonomics Association, “the scientific discipline concerned with the understanding of interactions among humans and other elements of a system, and the profession that applies theory, principles, data, and methods to design in order to optimize human well-being and overall system performance” [29]. More specifically, we draw on macroergonomics, the subdiscipline specializing in the design of organizations and work systems, with its strong theoretical grounding in sociotechnical systems (STS). STS theory arose from the Tavistock Institute after World War II, with a focus on improving work [30]. As indicated by the name, STS highlights that there are two subsystems: the social, consisting of people who sometimes work as teams, and the technical, including equipment, machines, tools, and technology, and these two subsystems interact within an internal environment that includes both the physical setting and organizational/managerial structure as well as the external environment [31,32]. The interactions between the people, technology, and environment shape how systems behave through emergent properties, which cannot be decomposed. Many approaches to understanding safety in STS have been described (see Carayon et al. [27] for a summary).

In this study, we use the work system model from macroergonomics [33,34,35], which is embedded in the Systems Engineering Initiative for Patient Safety (SEIPS) model [36,37,38,39]. These models posit that any work situation—where effortful activity is undertaken in pursuit of a goal, paid or unpaid—can be holistically described as composed of six work system elements (work system elements are italicized):

- The *team* of people who have their own unique skills, abilities, limitations, and characteristics.
- The *tasks* or goal-directed activities involved in pursuing the goal(s).
- The *tools and technologies* used in those tasks, including digital health technology, medical devices, paper and pencil, etc.
- The *physical environment* where the tasks are performed, which includes the physical layout, workstation design, noise, lighting, temperature, humidity, and air quality.
- The *organization* in which the work occurs, which includes both formal and informal organization, rules, procedures, management structures, climate, and culture.
- The *external environment* that includes rules, standards, and legislation outside of that particular organization as well as industry characteristics, like payment and reporting requirements in health care.

These work system elements interact and shape the processes involved in giving and receiving care (in care delivery settings) and activities that individuals take in pursuit of their own health in their homes and communities (e.g., patient work). These processes then result in outcomes for the patients, health care workers, and other formal and informal caregivers involved. The SEIPS model includes feedback loops, in which monitoring of system, process and outcome informs adjustment of the design of the work system—ideally, to bring about more positive outcomes. Importantly, culture is part of the organization’s work system element: culture helps to shape how work processes unfold over time.

The interactions between system components (i.e., work system elements) result in emergent properties of the system, which can positively or negatively influence the workers, work, and outcomes—these emergent properties must be inferred from examining the system components as a whole [40,41,42,43]. Some of these interactions have been studied as work system barriers and facilitators—factors that hinder or help the performance of work and achievement of goals [44,45,46,47]. Work system barriers/facilitators can arise from characteristics of individual work system elements or the interaction of two or more work system elements involved proximally—in other words, those that are involved most closely and immediately to create that barrier/facilitator. Other work system elements can be involved distally, indirectly resulting in the barrier/facilitator. For example, organizational culture may specify a specific method for a clinician to complete a task, requiring the use of a poorly designed technology that is difficult to use, hindering a clinician from completing that task. In this case, the barrier is proximally related to the design of the tool and technology but distally related to the organization. Of course, with the systems view of SEIPS, at some point all work system elements will be distally involved, and developing methods to conceptualize, describe, and evaluate these multi-level interactions and ‘system-ness’ remains an ongoing opportunity [27,38,48,49], albeit with slow but steady progress [50,51,52].

In this study, we use the SEIPS model [36,37,38] to investigate and describe how the sociotechnical system shapes safety culture in postpartum hemorrhage in an academic tertiary medical center. Given our focus on safety culture, we are particularly interested in how other elements of the work system interact with the organization’s work system element to impact culture.

## 2. Materials and Methods

This qualitative study is part of a larger project to design solutions to support the anticipation, detection, and response to maternal hemorrhage in prepartum, intrapartum, and postpartum care, aiming to ultimately reduce hemorrhage occurrence. This study is a secondary analysis of interview data [53]. The Institutional Review Board at Peoria determined that this study did not meet the definition of human subjects research.

### 2.1. Setting and Sample

The setting for this study is an obstetrics and gynecology (OB/GYN) department at an academic tertiary medical center in the Midwestern United States. The department staff includes 10 attending obstetrician physicians and obstetricians-in-training (i.e., resident physicians) each, 112 nurses, nine certified surgical technologists (CSTs) and 10 patient care technicians (PCTs). The year preceding data collection, delivery data at the participating medical center included 2259 deliveries and 261 hemorrhages (i.e., a hemorrhage rate of 11.6%). In these data, hemorrhages were defined as deliveries that were either coded as a hemorrhage and/or in which the patient had quantitative blood loss of >1000 mL (either or both of the previous) and that the patient received hemorrhage mitigation medications, underwent procedures, received transfusions, or had a predefined change in hematocrit. Hemorrhage procedures included uterine artery embolization, B-Lynch procedure, O’Leary stitch or uterine artery ligation, balloon compression (e.g., Bakri balloon), negative pressure system use (e.g., Jada device), suction dilation and curettage, or hysterectomy. Predefined changes in hematocrit included critically low values or significant change from baseline at admission for labor and delivery within 48 h of delivery.

We recruited 29 participants for this study, including five attending obstetricians, 11 obstetricians-in-training, 10 nurses, two CSTs, and one PCT between September and December 2023. The length of the experience of participants is shown in Table 1. We recruited participants in person and did not offer any incentive for participation. Participation was voluntary.

### 2.2. Data Collection Methods

We conducted semi-structured interviews in this study to allow follow-up probing for detailed responses [54]. The nurses, CSTs, and PCT were interviewed individually—due to scheduling constraints, the obstetricians and obstetricians-in-training were either interviewed individually or in small groups, separated by role.

Two researchers who were not affiliated with the OB/GYN department and thus did not have any authority over the participants led the interviews using an interview guide (the *Maternal Hemorrhage Interview Guide* is available at https://hfss.ise.illinois.edu/tools/, accessed on 17 February 2025). The questions included background information about the participant, the process and workflow of managing a postpartum hemorrhage across four phases of care, with probing questions about all elements of the work system, work system barriers and facilitators, and team formation and teamwork. One interviewer led each interview, while the other managed the logistics, kept time and took notes.

The interviews were conducted in person or via Zoom [55] and lasted an average of 39 min (total 12 h and 4 min). They were audio-recorded and transcribed.

### 2.3. Data Analysis Methods

The interview transcripts were reviewed for accuracy, and identifying details were removed. We uploaded each transcript to Dedoose (version 9.0.107) [56], a software used to manage qualitative and mixed methods analysis. As previously reported, we first performed a work system analysis and an inductive content analysis of the transcripts to identify all work system barriers and facilitators. Two researchers participated in the work system and barrier/facilitator analyses to enhance rigor; these results have been reported and identified 753 barriers/facilitators that were grouped into thirteen related groups: role ambiguity, anticipation, physical environment, staffing, resources/equipment, tools and technology, communication, coordination, cooperation, tacit knowledge, time pressure, leadership, and training [53].

In the current analysis, two researchers reviewed all individual barriers and facilitators to identify those related to safety culture. The barrier or facilitator was determined to be related to the safety culture if it reflected how organizational characteristics, such as the team culture, rules or processes, and management or leadership, influenced patient safety. We then inductively categorized those into broader dimensions and identified which work system element (or interaction between multiple elements) created those barriers or facilitators to positive safety culture.

We took special care to ensure rigor in this qualitative study [57]. We performed member checking to ensure credibility, clearly documented the context and methods to enhance transferability, and archived the iterations of our data analysis to enhance the dependability of our study [57,58]. Involving two researchers enhanced the credibility and confirmability of our work [57].

## 3. Results

**Figure 1:**
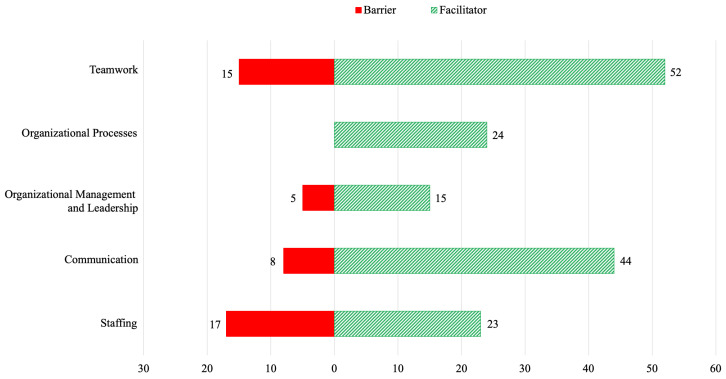
Count of work system barriers and facilitators by emergent property.

We inductively categorized the 45 barriers and 158 facilitators into five emergent properties related to the safety culture (i.e., staffing, communication, organizational management and leadership, organizational processes, and teamwork). Figure 1 depicts the number of work system barriers and facilitators related to each emergent property, while Table 2 lists examples of barriers and facilitators.

### 3.1. Staffing

Staffing refers to the availability of adequate and appropriate staff to manage the workload during postpartum hemorrhage events without feeling rushed (adapted from [22,59]). The proximally involved work system elements are organization, team, and tasks, as the organizational management determines the number of staff members assigned to complete tasks each shift. This emergent property was mentioned in 17 barriers and 23 facilitators.

In 16 barriers, participants described that it is challenging to complete patient care tasks promptly when the unit is short-staffed. In the remaining barrier, a participant stated that when working with team members who are inexperienced in responding to postpartum hemorrhage events, the more experienced team members have to assume multiple roles to ensure the patient is safe.

In 15 facilitators, participants noted that when adequate staff is present, they can proactively assess the patient for symptoms of postpartum hemorrhage and perform patient care tasks promptly. In five facilitators, participants mentioned that an attending physician or resident physician is frequently available to support patient tasks, enabling timely patient care. In three facilitators, participants stated that when the unit is short-staffed, clinicians from other teams, such as the maternal-fetal transport team, can be reassigned to the labor and delivery unit.

### 3.2. Communication

Communication refers to factors that impact open and closed-loop communication about errors, preventing errors, or ensuring patient safety during postpartum hemorrhage events (adapted from [22,60]). The proximally involved work system elements are tasks and organization. The tasks element is proximally related, as communication, or lack thereof, impacts the patient care tasks performed during postpartum hemorrhage events. The organization element is proximally related, as the organizational culture may impact whether clinicians communicate openly to prevent errors and understand how errors occur. This emergent property was mentioned in eight barriers and 44 facilitators.

In seven barriers, participants stated that there is commonly a lack of communication between team members during postpartum hemorrhage response. They cited reasons such as clinicians being flustered during an emergency and physicians not being physically present in the labor and delivery unit. In the remaining barrier, a participant described how when there are varying perceptions about the hemorrhage risk level, they feel they must convince their teammates of their patient safety concerns.

In 27 facilitators, participants stated that they feel encouraged and comfortable speaking up when they have a patient safety concern and that they think their teammates are sharing pertinent information. Seven facilitators were related to participants using closed-loop communication during postpartum hemorrhage events. In six facilitators, participants stated that having resident physicians as teammates facilitates communication since they frequently ask questions or communicate their decision-making process. The remaining four facilitators were related to the use of technology, specifically pagers, to communicate that help is needed for an ongoing hemorrhage situation.

### 3.3. Organizational Management and Leadership

This property refers to how organizational management and leadership impact patient safety during postpartum hemorrhage events (adapted from [22,39]). The proximally involved work systems elements are organization and team, as organizational management and the leader of the team may influence patient safety, such as commitment to patient safety or collaboration between interdisciplinary departments. This emergent property was mentioned in five barriers and 15 facilitators.

In all five barriers, participants stated that the leadership of the team negatively impacts patient safety, as poor leadership causes a high-anxiety situation and makes it difficult to share their clinical opinion. In 11 facilitators, participants mentioned that a leader with prior experience, good communication skills, and a calm presence is helpful during postpartum hemorrhage events. The remaining four facilitators were related to how organizational leadership promotes patient safety during postpartum hemorrhage events. Participants stated that the organizational leadership mandated annual training for all team members to ensure they were aware of best practices and established a quality assurance committee to review cases of postpartum hemorrhage to identify possible improvements in patient safety.

### 3.4. Organizational Processes

This property refers to how the organizational rules, processes, and procedures impact patient safety during postpartum hemorrhage events (adapted from [22,39]). The proximally involved work system elements are tasks and organization. The tasks element is proximally involved as the organizational processes directly impact which tasks are performed and the order in which the tasks are performed. The organization element is proximally involved as these rules, processes, and procedures are set at the organizational level. This emergent property was not mentioned in any barriers. However, it was mentioned in 24 facilitators.

In 18 facilitators, participants mentioned that they follow a general postpartum hemorrhage management process, outlined by the organization, that follows best practices published by the American College of Obstetricians and Gynecologists. The remaining six facilitators were related to the organizational process of performing a retrospective on postpartum hemorrhage events. During these retrospectives, clinicians are encouraged to discuss what occurred during postpartum hemorrhage management and identify areas of improvement.

### 3.5. Teamwork

Teamwork refers to how the organizational culture and climate impact the willingness of each clinician to work together during postpartum hemorrhage events (adapted from [22,60]). The proximally involved work system elements are team and organization. The team element is proximally involved since members must be willing, or want to, cooperate within the team. The organization element is proximally involved as the organizational culture and climate impact teamwork. This emergent property was mentioned in 15 barriers and 52 facilitators.

In ten barriers, participants stated that the personality of their team members may negatively impact teamwork during postpartum hemorrhage events. Some participants said that they sometimes avoid voicing concerns about patient safety as it sometimes creates tension between team members. Relatedly, in three barriers, participants mentioned that they often feel burnt out from the high demands experienced during postpartum hemorrhage management, which may negatively impact teamwork. In the remaining two barriers, participants mentioned that teamwork is negatively affected by the prioritization of charting over patient care tasks and a perceived lack of support for clinical roles such as surgical technicians.

In 52 facilitators, participants described their teammates as helpful, respectful, knowledgeable, and collaborative. They said their teammates recognize the seriousness of postpartum hemorrhage and are willing to help one another, regardless of their workload. Additionally, participants mentioned that the organizational culture supported their willingness to ask for help.

## 4. Discussion

In this study, we identified five emergent properties that contain 158 facilitators and 45 barriers related to safety culture (i.e., staffing, communication, organizational management and leadership, organizational processes, and teamwork). Our findings provide insight into how the sociotechnical system is related to safety culture, specifically during postpartum hemorrhage; unsurprisingly, given our focus on safety culture, the organization work system element was focal.

### 4.1. Proximally Involved Work System Elements

The five emergent properties were proximally related to three of the six work system elements. As described in the methods, a proximally involved work system element is the element (or interaction between multiple work system elements) that created the work system barrier or facilitator [38]. The team, tasks, and organization elements directly impact the safety culture. As highlighted previously, the organization element was proximally related to the five emergent properties. In comparison, the team element was related to one, and the tasks element was related to two properties. Considering our original definition of safety culture as the beliefs, values, and norms shared by members of the organization that influence their actions and behaviors related to and/or in pursuit of safety [22,23,24], this is unsurprising. The *organization* element includes the culture of the organization—as mentioned previously, safety culture is a specific aspect of the culture of an organization. The behaviors of people—the things they do—often are performed as part of the tasks the people complete in the organization, hence the relationship to the *task* element. Finally, the team is the people who have their own experiences and characteristics that also relate to beliefs and values [39,61]. In our study, the physical environment, external environment, and tools and technologies elements did not directly impact the safety culture involved in the care of postpartum hemorrhage. However, we would argue that these are distally related to culture and may emerge in future studies. For example, what an organization values—distinctly part of culture—can impact decisions in purchasing supplies or tools that can impact patient safety, such as investing in more hemorrhage carts to improve access in the case of a hemorrhage event. The organization’s values can also influence how the physical environment is designed in terms of light, layout and sound levels, which also influences the safety of patients and workers. Similarly, the design of technologies can change the likelihood of errors and harm [62], and the beliefs and values of an organization’s culture are likely to influence what technology they purchase and implement. The external environment, for example, the rules and regulations related to Medicare and Medicaid reimbursement, may influence the values of the organization. As described in the SEIPS model, changes to one or more work system element impacts the others, which in turn changes how the care processes unfold.

Our finding coincides with the predictors of safety violations identified by Alper and Karsh, who performed a systematic review of safety violations across multiple industries [63]. In this review, Alper and Karsh defined safety violations as non-malevolent actions (i.e., actions that were not intended to harm or damage the system) [63]. The authors identified six categories of predictors of safety violations: individual characteristics, information or training, design to support worker needs, safety climate, competing goals, and problems with rules [39,64]. Table 3 maps examples of these predictors of safety violations to the work system elements. As shown in Table 3, the predictors of safety violations are related to the team, tasks, tools and technologies, and organization elements—with the organization element being the most common. We expect that the tools and technologies element was not proximally related to the emergent properties, but instead, it was distally related (i.e., it creates the potential for a work system barrier or facilitator but is not the immediate cause) [38]. This expectation is supported by the four facilitators in the Communication property, in which the participants mentioned that they use pagers to communicate that help is needed for an ongoing hemorrhage situation. While the physical and external environments were not highlighted in our findings, given that all elements of the work system shape processes and outcomes, our assumption is that these elements could be involved, and alternative methods (e.g., observation) might uncover the impact of the physical environment or the involvement of other stakeholders in other levels of the organizational leadership, but not involved in the direct provision of care, might identify the impact of rules and regulations outside the organization.

### 4.2. Strategies to Improve Safety Culture

Overall, our study participants identified more positive aspects than negative related to safety culture, perhaps suggesting that they experience a positive safety culture currently, which positively influences their actions and behaviors. However, the negative aspects indicate that safety culture, the process involved in postpartum hemorrhage care, and potentially patient outcomes could be improved by redesigning the work system to mitigate the barriers.

Work system redesign using the SEIPS model has been performed in many empirical studies spanning several health care settings. For example, the SEIPS model has been used to analyze existing work systems in nursing homes [65,66], care transitions [59,67,68,69,70], and inpatient hospitals [71]. The SEIPS model has also been used to identify work system design principles and interventions in nursing homes [72] and pharmacies [73,74]. Results of a work system analysis can then be used to design and develop interventions [75], ensuring fit with the rest of the work system to avoid disrupting workflow [76] and creating negative, unanticipated consequences [50]. In this study, social-based interventions seem to be highlighted—having adequate staff to respond to a hemorrhage, especially to allow one experienced clinician familiar with policies at this health care system, facilitated a swift, positive response to the hemorrhage and was indicative of a positive safety culture. So, hospital management might ensure that an experienced nurse—perhaps a charge nurse—is empowered to step away from charge duties, perhaps delegating to an individual of their choosing or someone already designated and helping to organize a hemorrhage response. Of course, delegating charge duties to another nurse would involve a handoff and lead to negative consequences on other work activities. From a technology perspective, a checklist shown on a shared, smart display, updated either automatically (based on sensors) or by a designated individual in the hemorrhage response, that shows the suggested workflow and role responsibilities during a hemorrhage response could support teamwork, communication, and awareness of suggested organizational processes. In developing and implementing these solutions, one could close the feedback loop, allowing safety culture to inform the design of the work system as well as the design of the work system shaping safety culture.

Outside of SEIPS-based analysis, other systems engineering techniques could be leveraged to facilitate hemorrhage response specifically or safety culture more generally [77]. For example, Lean Thinking could help to ensure the availability of resources (including staff) and thus improve safety culture. Lean Thinking, developed by the Toyota Motor Corporation as a strategy to optimize auto manufacturing in the United States, consists of concepts, methods, and tools to eliminate unnecessary waste and minimize delays in work [78,79]. Lean Thinking is commonly implemented in EDs to mitigate similar obstacles to those identified in our study (e.g., delays in providing care, difficulties in communication, and poor patient safety) [78,79,80,81,82]. Several empirical articles demonstrate significant improvements in patient outcomes after Lean Thinking Principles have been implemented. For example, in 2011, Holden performed a literature review of 18 empirical studies of EDs that implemented Lean Thinking. He found that implementing Lean Thinking in EDs led to various care process changes, such as staffing changes and new technologies and communication systems to support patient care [78]. A similar systematic literature review study was performed in 2021 by Souza and colleagues. They found that implementing Lean Thinking in EDs led to reductions in waiting time, patient flow, and procedure times, as well as improvements in efficiency, productivity, and patient safety [83]. Examples of Lean Principles that were implemented in the ED to mitigate barriers similar to the barriers identified by our participants include:

- Staffing Shortages: Division of medical and nursing staff to work on specific patient streams [78,84,85,86], altering staffing schedules to reassign physicians and nurses to match peak patient volume [78,87], and redefining responsibilities of medical and nursing staff [78,88].
- Communication and Teamwork: Implementing communication tools [78,87].

## 5. Conclusions

In this study, we explored how factors related to safety culture help and hinder the response to postpartum hemorrhage—a leading cause of maternal mortality in the United States and worldwide. Adequate staffing, adequate and efficient communication, organizational management and leadership, clear and salient organizational processes, and good teamwork were all related to positive safety culture in hemorrhage responses. Additional opportunities, targeting both social and technical subsystems of the sociotechnical system in postpartum hemorrhage response, could build upon these results. Ultimately, our study provides additional empirical evidence that the design of the sociotechnical system shapes safety culture—and closing feedback loops by designing, developing, and implementing sociotechnical-based solutions shows how safety culture can shape the sociotechnical system.

This study does indeed have some limitations. As highlighted in the discussion, triangulating multiple data streams (e.g., including observation) and/or interviewing additional stakeholders in the organization who do not directly provide care but do influence hemorrhage response processes might uncover additional emergent properties that influence safety culture. Further, including some review of actual patient outcomes via chart review and a survey-based measure of safety culture would have helped to gain a deeper understanding of the safety culture at this site. Importantly, we did not interview any patients, who would have important insights into safety culture and should be included in the future. Lastly, this study aimed to focus on postpartum hemorrhage at one participating health care site—our findings may not generalize to other sites, and a careful comparison of contexts is needed.

## Figures and Tables

**Table 1:** Duration of the experience of participants in positions responding to postpartum hemorrhage.

Role	Number of Participants (n)	Average Experience (Years)	Range of Experience (Years)
Attending Obstetricians	5	8.8	2–18
Obstetricians-In-Training	11	1.86	0.5–3.5
Nurses	10	8.90	1–18
Certified Surgical Technologists	2	11.75	3.5–20
Patient Care Technicians	1	41	41

**Table 2:** Examples of barriers and facilitators in each emergent property.

Emergent Property	Proximally Involved Work System Element(s) *						Example
	Tm	T	TT	O	PE	EE	
Staffing	X	X		X			Barrier: *“I would say on the days when we are short-staffed. Everybody, it’s almost like they have tunnel vision. They’re so busy doing their own job that it’s, you’re, you’re so busy. You’re not seeing the full picture. Um, and it’s very hard to work together because you just, like I got to get my job done, I got to get my stuff done.”* (Labor and Delivery RN) Facilitator: “*Usually multiple nurses will respond as well, including like the charge nurse if it’s becoming serious enough. So we have lots of hands, lots of help.”* (Resident Physician)
Communication		X		X			Barrier: *“I think sometimes people just get flustered in emergency situations and it’s hard to, for everyone to communicate.”* (Labor and Delivery RN) Facilitator: “*I think, um, everyone is like comfortable with each other for the most part and is, feels comfortable like communicating concerns.”* (Labor and Delivery RN)
Organizational Management and Leadership	X			X			Barrier: *“I’m always happy to be working with a nurse that has, years and years more experience than I do. I always feel more confident that way. Um, so it’s generally a positive thing…I think that, um, it’s something that [residents must learn], is how to navigate that. Because, um, you know, if they, for example, disagree with you on something, what do you do next?”* (Attending Physician) Facilitator: *“I think that there is a recognition that this is important. So we have regular mandated simulation days at least once a year regarding about, postpartum hemorrhage and what to do, and that’s required for all attendings, residents and nursing staff and patient care techs. So once a year we do go to those and they focus on keeping that up to date, up to date. And we address also any hemorrhage incidents often in our QA committee. So we have a QA committee that reviews cases and if there’s any significant findings, we address them and we talk about them there.”* (Labor and Delivery RN)
Organizational Processes		X		X			Barrier: None. Facilitator: *“I mean there are like policies and procedures so that you can recognize it early and follow and follow the right algorithm to make the bleeding stop. Is anybody going to pull it off the computer at that particular moment? No, we just kind of know that A, B, and C are happening and we need to go…We need to address all of these and maybe go to D. And I mean, there’s, I think the hemorrhage policy is posted places.”* (Labor and Delivery RN)
Teamwork	X			X			Barrier: *“Well, it (the culture) would depend on, um, who was what we call the doc in the box. Um, that is like, or like laborist is what they call them. Um, there has to be somebody over on labor 24/7. So it would just depend on who was on that day. Um, you know, their demeanor, how they come in, um, somebody might be a little bit more high strung. Somebody might be a little bit more chill coming in, you know? Um, I think that depends. You know who’s there, then the residents would come in, and again, it depends on personalities, you know who’s coming in. And then also the labor nurses, they pretty much come in and just kind of take over, you know, everything but that’s their cup of tea, you know. So, I think it just depends on more personalities.”* (Certified Surgical Technologist) Facilitator: *“[We] oftentimes are working in very close-knit situations and we have each other’s back and, um, take care of each other…We kind of have the opinion that all the patients on the floor are all of our patients and we just kind of help each other, especially like during emergencies and things like that.”* (Labor and Delivery RN)

* Note: Tm = team, T = tasks, TT = tools and technology, O = organization, PE = physical environment, EE = external environment.

**Table 3:** Mapping between work system elements and predictors of safety violations.

Work System Element	Definition [33,35,37,39]	Predictors of Safety Violations [63]
Person/Team	The individual or team (e.g., a group of individuals), at the center of the system	Individual attitude toward compliance with safety practices
Tasks	The tasks performed by the team. The tasks may vary by content and variety and may have varying characteristics (e.g., physical and psychological demands)	Competing tasks or goals
Tools and Technologies	The tools and technologies used to perform tasks. These tools and technologies can be simple, e.g., paper, or complex (e.g., electronic health record)	Inadequate tools
Organization	The formal and informal organization, organizational culture and climate, and the leadership and management	Training related to safety practices Staffing levels Management attitude toward compliance with safety practices Outdated rules or procedures
Physical Environment	The physical layout or design (e.g., workstation design, noise, lighting, etc.)	None
External Environment	Extra-organizational factors that may influence policy, standards, or characteristics of the health care domain	None

## Data Availability

The data presented in this study are available on request from the corresponding author due to participant confidentiality concerns.

## Abbreviations

The following abbreviations are used in this manuscript:

SEIPS	Systems Engineering Initiative for Patient Safety
STS	Sociotechnical system
OB/GYN	Obstetrics and gynecology
CST	Certified surgical technologists
PCT	Patient care technicians
